# Identification of Sp1 as a Transcription Activator to Regulate Fibroblast Growth Factor 21 Gene Expression

**DOI:** 10.1155/2017/8402035

**Published:** 2017-03-29

**Authors:** Shuqin Chen, Huating Li, Jing Zhang, Shan Jiang, Mingliang Zhang, Yilan Xu, Kun Dong, Ying Yang, Qichen Fang, Weiping Jia

**Affiliations:** Shanghai Diabetes Institute, Shanghai Key Laboratory of Diabetes Mellitus, Shanghai Clinical Center for Diabetes, Department of Endocrinology and Metabolism, Shanghai Jiao Tong University Affiliated Sixth People's Hospital, Shanghai, China

## Abstract

Fibroblast growth factor 21 (FGF21) is a metabolic hormone with multiple beneficial effects on lipid and glucose homeostasis. Previous study demonstrated that FGF21 might be one of the Sp1 target genes. However, the transcriptional role of Sp1 on FGF21 in adipose tissue and liver has not been reported. In this study, we found that the proximal promoter of mouse FGF21 is located between −63 and −20 containing two putative Sp1-binding sites. Sp1 is a mammalian transcription factor involved in the regulation of many genes during physiological and pathological processes. Our study showed that overexpression of Sp1 or suppressing Sp1 expression resulted in increased or reduced FGF21 promoter activity, respectively. Mutation analysis demonstrated that the Sp1-binding site located between −46 and −38 plays a primary role in transcription of FGF21. Electrophoretic mobility shift assay and chromatin immunoprecipitation analysis indicated that Sp1 specifically bound to this region. Furthermore, the binding activity of Sp1 was significantly increased in adipose tissues of HFD-induced obese mouse and liver of DEN-treated mouse. Thus, our results demonstrate that Sp1 positively regulates the basal transcription of FGF21 in the liver and adipose tissue and contributes to the obesity-induced FGF21 upregulation in mouse adipose tissue and hepatic FGF21 upregulation in hepatocarcinogenesis.

## 1. Introduction

Fibroblast growth factor 21 (FGF21), an atypical member of the FGF family, functions as a hormone that coordinates lipid and glucose metabolism through binding of the FGF receptors in the presence of the coreceptor *β*-klotho [1–5]. Transgenic mice with overexpression of FGF21 are resistant to diet-induced obesity and metabolic disturbance [2]. Treatment of genetic and diet-induced obese mice with pharmacologic doses of FGF21 leads to an improved metabolic profile, including decreased hepatic triglycerides, plasma triglycerides, and glucose levels [2, 6, 7].

Under physiological conditions, the fasting-induced FGF21 expression is mediated by the peroxisome proliferator-activated receptor alpha (PPAR*α*) in the liver [8, 9]. Increased hepatic FGF21 acts as an endocrine hormone to coordinate the adaptive starvation responses including gluconeogenesis and ketogenesis [8, 9]. In the fed state, studies reported that FGF21 is induced by the peroxisome proliferator-activated receptor gamma (PPAR*γ*) in the white adipose tissue (WAT), and FGF21 acts in an autocrine or paracrine manner to stimulate the activity of PPAR*γ*, a master transcriptional regulator of adipogenesis [10, 11]. Many studies found that FGF21 expressions are increased in liver and WAT in both diet-induced obese mice and genetically obese* db*/*db *and* ob*/*ob *mice [12–14]. Although elevated endogenous FGF21 fails to induce the desired physiologic effects of lipid and glucose homeostasis during obesity, pharmacological administration of FGF21 appears to exert beneficial actions to improve metabolic parameters in mice [6, 7]. These observations also imply that supraphysiological doses of FGF21 might be required for the treatment of obesity. Two recent clinical studies demonstrated that FGF21 analogs improve the lipid profile of obese and diabetic patients [15, 16], but the application of the FGF21 analogs is limited by the route of administration. Therefore, induction of FGF21 expression could be a candidate therapeutic regiment for the treatment of obesity and its associated metabolic disorders.

A previous study identified that FGF21 might be a target gene of Sp1 by functional genomics [17]. The experiment was performed in human Hela cells and the study focused on finding novel Sp1 targets involved in proliferation and cancer. However, the specific Sp1 binding sites in mouse FGF21 promoter and the transcriptional role of Sp1 in liver and in adipose tissues which are the predominant sources of FGF21 remain unknown. In our study, we characterized the promoter activity of mouse FGF21 gene in HepG2 cells and 3T3-L1 preadipocytes. Furthermore, we identified Sp1 contributes to the obesity-induced FGF21 upregulation in mouse adipose tissue and hepatic FGF21 upregulation in hepatocarcinogenesis.

## 2. Methods and Materials

### 2.1. Animals

All animal experimental procedures were carried out in accordance with the Guide for the Care and Use of Laboratory Animals of the Shanghai Jiao Tong University. Male C57BL/6J mice were purchased from Shanghai Laboratory Animal Co. Ltd. To generate diet-induced obesity model, mice at 6 weeks of age were fed a high-fat diet (HFD) containing 60% kcal of fat or a normal diet (ND) for 12 weeks (Research Diets Inc.). Mice liver tissues of Diethylnitrosamine- (DEN-) induced hepatocarcinogenesis were from Shanghai Cancer Institute. Male C57BL/6J mice at 2 weeks of age were injected with DEN intraperitoneally (IP) at 10 mg/Kg body weight for 4 months. Control group was injected IP with PBS correspondingly.

### 2.2. Plasmids

The 5′-flanking region of mouse FGF21 gene (−1432 to +70 relative to the transcription start site) was amplified by polymerase chain reaction (PCR) using the BAC clone as a template. The PCR product was inserted into the luciferase reporter vector pGL3-Basic (Promega) to create construct p(−1432 to +70)Luc using restriction sites KpnI and BglII. The p(−1432 to +70)Luc was then used as the template to generate a series of 5′ deletion reporter gene constructs by using primers with KpnI and BglII sites at their ends. The substitution mutant constructs of the putative transcription factor binding sites were generated by the QuikChange site-directed mutagenesis (Stratagene). The nucleotide sequences of the primers used for various deletions and mutant constructs are listed in Table 1. All constructs were verified by DNA sequencing.

### 2.3. Cell Culture, Transient Transfection, and Luciferase Assays

The human hepatoma cell line HepG2 cells and 3T3-L1 preadipocytes were maintained in minimum essential medium or Dulbecco's modified Eagle's medium supplemented with 10% fetal bovine serum, 100 units/mL penicillin, and 100 *μ*g/mL streptomycin (Invitrogen). Cells were plated at 1 × 10^5^ cells/well in 24-well plates and precultured for 24 h. Transfection was performed with Lipofectamine 2000 reagent (Invitrogen) following the manufacturer's protocol. The cells were cotransfected with 0.3 *μ*g of various firefly luciferase reporter plasmids and 0.03 *μ*g* Renilla* luciferase-expressing plasmid (pRL-TK, Promega) using 1 *μ*L Lipofectamine 2000 reagent (Invitrogen) following the manufacturer's protocol. Forty-eight hours after transfection, cells were harvested and luciferases were assessed using the Dual-Luciferase Assay System (Promega) according to manufacturers' protocols. The Sp1 expression plasmid was kindly provided by Dr. Jianping Ye (Louisiana State University). The Sp1 shRNA plasmid and scramble control were generated previously [18]. For overexpression experiments, cells were cotransfected with reporter plasmids, pRL-TK, and Sp1 expression plasmid or the control vector using Lipofectamine 2000 reagent. Forty-eight hours later, luciferase reporter assay was performed according to the manufacturer's protocols. For RNA interference experiments, reporter plasmids, pRL-TK, and Sp1 shRNA plasmid or scramble control were cotransfected into the cells using Lipofectamine 2000 reagent. Forty-eight hours after transfection, luciferases were assessed according to the manufacturer's protocols.

### 2.4. Electrophoretic Mobility Shift Assay (EMSA)

Double-stranded oligonucleotides used as probes and competitors in this study were synthesized (Table 1). The probe of FGF21 containing the region between −51 to −31 includes the Sp1-B binding site. The probe of Sp1C represents promoter of DsbA-L gene containing the consensus Sp1-binding site [18]. Nuclear extracts of mice hepatocytes and adipocytes were prepared using NE-PER nuclear and cytoplasmic extraction reagents (Pierce) according to the manufacturer's instructions. Protein concentration was determined by the bicinchoninic acid (BCA) assay and the nuclear extracts were stored at −80°C. The DNA-protein binding reactions were performed in a 20 *μ*L reaction mix using the LightShift EMSA Optimization and Control Kit (Pierce). In brief, nuclear extracts were preincubated in reaction mix containing 1x binding buffer, 2.5% glycerol, 5 mM MgCl_2_, 1 *μ*g of poly(deoxyinosinic-deoxycytidylic) [poly(dI-dC)], and 0.05% NP-40. After 5 min at room temperature (25°C), 20 fmol of labeled probes was added and incubation was continued for another 20 min. Protein–DNA complexes were separated from the free probe by electrophoresis on a 5% native polyacrylamide gel in 0.5x Tris-Borate-EDTA (TBE) buffer. After electrophoresis, the DNA-protein complexes were transferred to a nylon membrane and cross-linked at 120 mJ/cm^2^ using a CL1000 Ultraviolet Crosslinker (UVP). Biotin-labeled DNA was detected by Chemiluminescent Nucleic Acid Detection Module (Pierce) and ImageQuant LAS 4000 mini (GE Healthcare). For the competition binding reactions, the unlabeled competitor in molar excesses of the labeled probe was included in the reaction for 5 min prior to the addition of the labeled probes.

### 2.5. Chromatin Immunoprecipitation (ChIP)

ChIP assays were performed by using the Agarose ChIP Kit (Pierce) according to the manufacturer's instructions. Liver and adipose tissues were subjected to cross-linking with 1% formaldehyde. After stopping the reaction with 0.1 M glycine, the chromatin was sheared into fragments of 500–1000 bp in length and immunoprecipitated with antibody against Sp1 (sc-59x, Santa Cruz) or negative control immunoglobulin G (IgG) provided by this kit overnight at 4°C. Bound DNA was purified and amplified by PCR with primers (forward 5′-TCCTGCCAAGTGTGTCAAAT-3′ and reverse 5′-GTGAACGCAGAAATACCAGAA-3′) which amplified the region of the FGF21 promoter containing target Sp1-B binding site. The PCR products then were separated on 2% agarose gels.

### 2.6. Quantitative Real-Time PCR Analysis

Total RNAs were extracted from liver and adipose tissue by Trizol reagent (Invitrogen). Real-time PCR was performed using the SYBR Green PCR Master Mix (Applied Biosystems). GAPDH was used as an endogenous control. The primers used for detecting expression of FGF21 were 5′-CTGCTGGGGGTCTACCAAG-3′ and 5′-CTGCGCCTACCACTGTTCC-3′.

### 2.7. Western Blot Analysis

Antibody against Sp1 was from Santa Cruz Biotechnology (sc-59). Anti-*β*-actin antibody was from Abcam (ab6276). Anti-*β*-tubulin antibody was from Sigma-Aldrich (T8328). Total protein was extracted from liver or adipose tissues by RIPA buffer supplemented with protease inhibitor cocktail and phosphatase inhibitors (Sigma). Protein was separated by SDS-PAGE and transferred to polyvinylidene difluoride membrane (Millipore). After incubation with the desired antibodies, the blots were developed with Immobilon Western Chemiluminescent HRP substrate (Millipore).

### 2.8. Statistical Analysis

All data were analyzed by Student's *t* tests or ANOVA, followed by Dunnett post hoc test using GraphPad Prism (GraphPad Software). *P* values <0.05 were considered statistically significant.

## 3. Results

### 3.1. Sequence Analysis of FGF21 5′-Flanking Region and Identification of Mouse FGF21 Proximal Promoter

**Figure 1:**
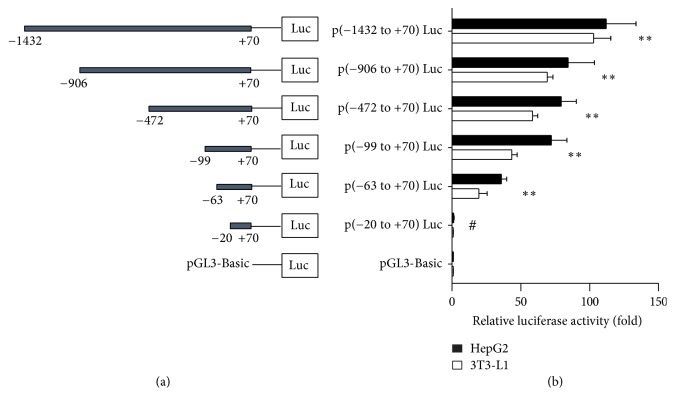
Deletion analysis of FGF21 promoter in HepG2 and 3T3-L1 cells. Schematic diagram of reporter constructs containing the indicated promoter fragments of the mouse FGF21 gene is shown on (a). The numbering is relative to the transcription start site. The corresponding luciferase reporter assay results are shown at (b). The relative promoter activity is expressed as the ratio of each construct to the promoterless control pGL3-Basic. Data represent the mean ± SD of triplicate assays in three independent experiments. ^*∗∗*^*P* < 0.01 versus pGL3-Basic, ^#^*P* < 0.05 versus p(−63 to +70)Luc, p(−99 to +70)Luc, p(−472 to +70)Luc, p(−906 to +70)Luc, and p(−1432 to +70)Luc.

The mouse FGF21 promoter sequence was obtained from the National Center for Biotechnology Information (GeneBank accession number NC_000073.6). For identification of the proximal promoter region, a series of 5′ deletion promoter reporter constructs with a common 3′-terminus at nucleotide +70 were generated and transiently expressed into HepG2 cells or 3T3-L1 cells. Promoter activity was assessed by measuring luciferase activities. In 3T3-L1 cells, the p(−1432 to +70)Luc showed a 100-fold increase in promoter activity compared with the luciferase reporter vector pGL3-Basic (Figure 1). Deletion from −1432 to −906, −472, −99, and −63 [p(−906 to +70)Luc, p(−472 to +70)Luc, p(−99 to +70)Luc, and p(−63 to +70)Luc] resulted in a stepwise decrease of promoter activity. However, further deletion to −20 [p(−20 to +70)Luc] dramatically reduced the luciferase levels to about 4% of that of the p(−63 to +70)Luc, suggesting that the region located between −63 and −20 contributes to the basal activity of the FGF21 promoter. Similar results were observed in HepG2 cells.

### 3.2. Putative Sp1-Binding Sites in the Proximal Promoter Are Essential for the Mouse FGF21 Gene Transcription

**Figure 2:**
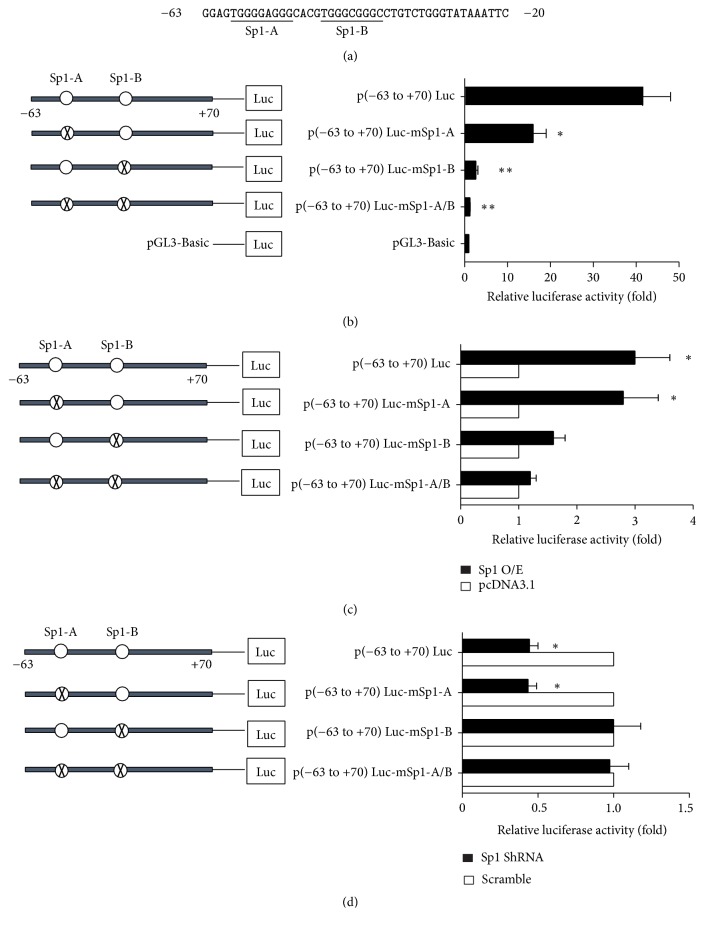
Mutation analysis of the predicted Sp1-binding sites. (a) Nucleotide sequence encompassing the Sp1-binding sites in FGF21 gene promoter. The numbering is relative to the transcription start site. The potential Sp1-binding sites predicted by MAPPER software are underlined. (b) Luciferase reporter assay of Sp1-mutant constructs in 3T3-L1 cells. The relative promoter activity is expressed as the ratio of each construct to the pGL3-Basic. ^*∗*^*P* < 0.05 versus p(−63 to +70)Luc, ^*∗∗*^*P* < 0.01 versus p(−63 to +70)Luc. (c) and (d) Analysis of the influence of Sp1 on FGF21 promoter activity. 3T3-L1 cells were cotransfected with the wild-type and Sp1-mutant reporter constructs and Sp1 expression construct or Sp1 shRNA plasmid. The relative promoter activity is expressed as the ratio of each construct to the vector control pcDNA3.1 or scramble control. ^*∗*^*P* < 0.05 versus pcDNA3.1 or scramble. Sp1-binding sites are indicated with open circles. The mutations introduced into the Sp1-binding sites are represented as circles with the cross. All data represent the mean ± SD of triplicate assays in three independent experiments.

To elucidate the mechanisms underlying the regulation of the basal transcriptional activity of FGF21 promoter, we searched for the transcription factor binding sites using the online software (MAPPER). As shown in Figure 2(a), two putative Sp1-binding sites, Sp1-A and Sp1-B, were found between −63 and −20, the region involved in regulating the basal promoter activity. To evaluate the contribution of these two putative binding sites to the regulation of FGF21 promoter, mutations of Sp1-binding sites were introduced into p(−63 to +70)Luc, creating mutant constructs p(−63 to +70)Luc-mSp1-A, p(−63 to +70)Luc-mSp1-B, and p(−63 to +70)Luc-mSp1-A/B. As depicted in Figure 2(b), mutation of Sp1-A [p(−63 to +70)Luc-mSp1-A] or Sp1-B [p(−63 to +70)Luc-mSp1-B] reduced FGF21 promoter activities to about 40% and 5% of the wild-type promoter activity, respectively. Double Sp1-binding sites mutation [p(−63 to +70)Luc-mSp1-A/B] reduced the promoter activity to about 3% of the wild-type promoter activity. These results indicated that Sp1-A and Sp1-B were positive regulator elements in the basal transcription of FGF21.

To further investigate the roles of these two Sp1-binding sites, Sp1 was overexpressed in 3T3-L1 cells transiently transfected with p(−63 to +70)Luc or with the Sp1-mutant constructs (Figure 2(c)). The promoter activity of the wide-type construct p(−63 to +70)Luc was stimulated to 3-fold by overexpression of Sp1. However, the promoter activities of p(−63 to +70)Luc-mSp1-B and p(−63 to +70)Luc-mSp1-A/B could only be induced to 1.5-fold and 1.1-fold, respectively. Interestingly, p(−63 to +70)Luc-mSp1-A could also be stimulated to 2.8-fold, which was comparable to that of the wide-type p(−63 to +70). In contrast, suppressing Sp1 expression by siRNA significantly reduced the promoter activities of p(−63 to +70)Luc and p(−63 to +70)Luc-mSp1-A by about 56% compared to the scramble groups (Figure 2(d)). However, the promoter activities of p(−63 to +70)Luc-mSp1-B and p(−63 to +70)Luc-mSp1-A/B were not affected. Taken together, the results suggested that Sp1-B was the core binding site in the transcription of FGF21 gene.

### 3.3. Sp1 Specifically Binds to the Proximal Promoter of the Mouse FGF21 Gene

**Figure 3:**
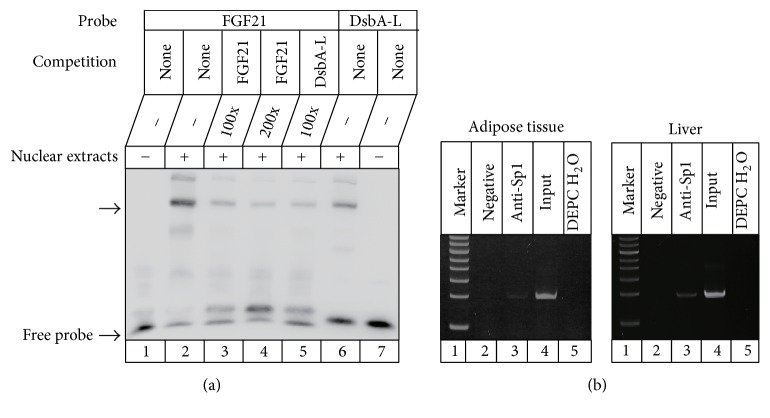
Specific binding of Sp1 to the FGF21 promoter. (a) EMSA assay. Nuclear extracts from adipose tissue were incubated with biotin-labeled FGF21 probe encompassing the putative Sp1-B binding site, and Sp1C-probe containing the consensus site for Sp1. Competition experiments were performed using 100-fold and 200-fold excess of the unlabeled FGF21 oligonucleotide. Cross competition was performed using a 100-fold excess of the unlabeled Sp1C oligonucleotide. The specific DNA-protein complexes and free probes are indicated by the arrow. (b) ChIP analysis. Formaldehyde-crosslinked chromatin from adipose tissue or liver was incubated with anti-Sp1 antibody. As the negative control, the chromatin was incubated with nonspecific IgG. DNA immunoprecipitated using the antibody against Sp1 was analyzed by PCR with primers specific for the FGF21. Input DNA and diethylpyrocarbonate-treated water (DEPC H_2_O) were used as a positive or negative control for the PCR reaction, respectively.

To determine the interaction of Sp1 with the putative binding site, we performed EMSA. Since our aforementioned data had demonstrated that Sp1-B play a primary role in the transcription of FGF21 gene, the oligonucleotide used as the probe in EMSA only bears the Sp1-B site. The oligonucleotide formed a prominent complex with the nuclear extracts from adipose tissue (Figure 3(a), line 2). The DNA-protein complex had mobility similar to that of the complex with the Sp1C probe (line 6), which contains the consensus Sp1-binding site and has a similar core sequence to that of the potential Sp1-B binding site in the FGF21 promoter. The formation of the complex of FGF21 probe could be competed using excess of the unlabeled FGF21 probe (lines 3, 4) or unlabeled Sp1C probe (line 5). Similar results were observed using the nuclear extracts from liver (data not shown). To further confirm the direct binding of Sp1 to the FGF21 promoter in vivo, we performed ChIP assays using C57/BL mice adipose tissue and liver (Figure 3(b)). Anti-Sp1 antibody immunoprecipitated protein–DNA complexes were recovered and the purified DNA was used as a template for PCR using primers covering the potential Sp1-B binding site. A 93-bp PCR product was amplified from the DNA fragment immunoprecipitated by the anti-Sp1 antibody (Figure 3(b), lane 3). Collectively, these results indicated that Sp1 specifically bound to the binding site within the region of the FGF21 promoter in vitro and in vivo.

### 3.4. Sp1 Is Responsible for the Upregulation of FGF21 Expression in Adipose Tissue of Diet-Induced Obesity Mice

**Figure 4:**
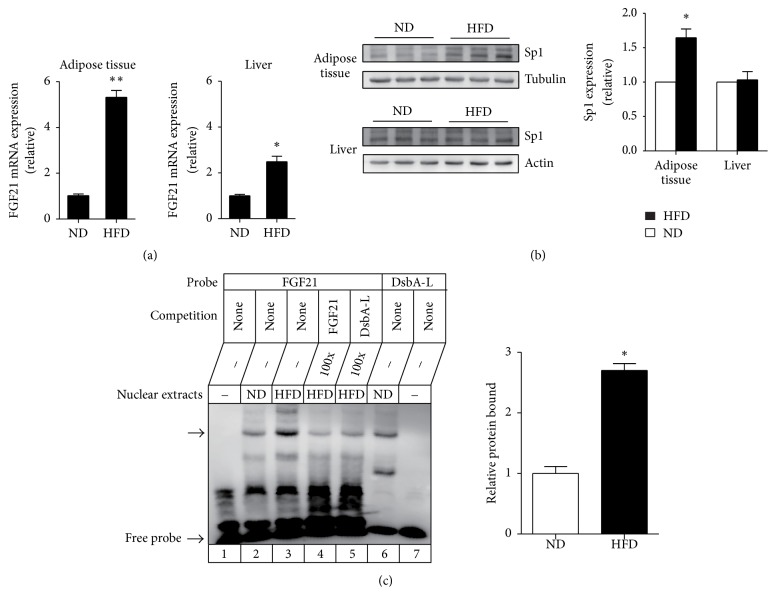
Sp1 is responsible for the upregulation of FGF21 in adipose tissues of diet-induced obese mice. (a) Real-time PCR analysis of FGF21 expression in the adipose tissue and liver of C57BL/6J mice fed with normal diet (ND) or high-fat diet (HFD). Data are means ± SEM, *n* = 5. ^*∗*^*P* < 0.05 versus ND-fed mice. ^*∗∗*^*P* < 0.05 versus ND-fed mice. (b) Western blot analysis of Sp1 protein expression in the adipose tissue and liver of C57BL/6J mice fed with ND or HFD. Quantification of the relative protein levels (expressed as the percentage of ND-fed mice protein level, arbitrarily set as 1.0) was performed by analyzing Western blot data from three independent experiments using the Scion Image program. Tubulin or actin was used as a loading control. Data are means ± SEM, *n* = 5. ^*∗*^*P* < 0.05 versus ND-fed mice. (c) EMSA assay. Nuclear extracts from adipose tissue of ND or HFD-induced mice were incubated with the biotin-labeled probe containing the sequence of the Sp1-B binding site. Competition experiments were performed using 100-fold unlabeled FGF21 oligonucleotide. Cross competition was performed using 100-fold excess unlabeled Sp1C oligonucleotide. The specific DNA-protein complexes and free probes are indicated by the arrow. Quantification of the relative change in protein bound (expressed as the percentage of ND-fed mice protein bound, arbitrarily set as 1.0) was performed as described in (b). ^*∗*^*P* < 0.05 versus ND-fed mice.

Previous studies have demonstrated that FGF21 mRNA level was markedly increased in the liver and adipose tissue of* db*/*db *or diet-induced obesity mice [13, 19]. Given that Sp1 positively regulates FGF21 basal transcriptional activity in HepG2 cells and 3T3-L1 cells, we investigated whether Sp1 has a direct effect on the upregulation of FGF21 transcription in HFD-induced obesity mice model. Consistent with other studies, our results show that HFD feeding significantly increased the FGF21 mRNA expression in both liver and adipose tissues (Figure 4(a)). At the same time, the protein level of Sp1 was increased in adipose tissue but not in the liver of HFD-fed mice (Figure 4(b)), implying that Sp1 may be responsible for the HFD-induced FGF21 upregulation in adipose tissue. So we further performed EMSA to demonstrate the Sp1 binding activity in adipose tissues of diet-induced obesity mice. As shown in Figure 4(c), the intensity of DNA-protein complex was markedly increased in adipose tissues of HFD-fed obese mice compared with ND-fed mice, which was consistent with the results of the Sp1 expression. These results suggested that Sp1 may be responsible for the upregulation of FGF21 expression in adipose tissue of diet-induced obesity mice.

### 3.5. Sp1 Can Induce the Upregulation of FGF21 in Liver of DEN-Treated Mice

**Figure 5:**
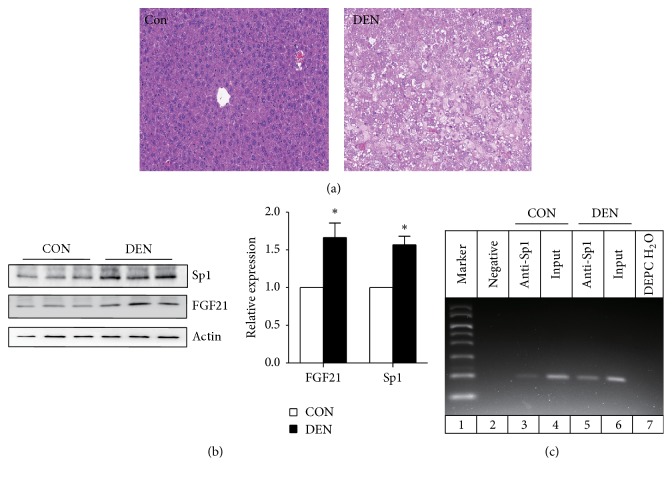
Sp1 can induce the upregulation of FGF21 in liver of DEN-treated mice. (a) Histochemical analysis of liver samples from mice treated with DEN or PBS. Liver tissues were collected at 4 months after injection and then fixed overnight in 4% PFA in 1x PBS. Paraffin-embedded tissue blocks were sectioned into 5 *μ*m slides for H&E staining. (b) Western blot analysis of FGF21 and Sp1 protein levels in the liver of C57BL/6J mice injected with PBS or DEN for 4 months. Quantification of the relative protein levels (expressed as the percentage of PBS-treated mice protein level, arbitrarily set as 1.0) was performed by analyzing Western blot data from three independent experiments using the Scion Image program. Actin was used as a loading control. Data are means ± SEM, *n* = 5. ^*∗*^*P* < 0.05 versus PBS-treated mice. (c) ChIP analysis. Formaldehyde-crosslinked chromatin from liver of C57BL/6J mice treated with PBS or DEN was incubated with anti-Sp1 antibody. DNA immunoprecipitated using the antibody against Sp1 was analyzed by PCR with primers specific for the FGF21. Input DNA were used as a positive control for the PCR reaction. The chromatin incubated with nonspecific IgG and diethylpyrocarbonate-treated water (DEPC H_2_O) were used as negative control.

We found that previous studies had reported that FGF21 is induced in response to chemical (DEN treatment) and genetic-induced hepatocarcinogenesis [20, 21]. Therefore, we aim next to investigate whether the increased expression of FGF21 in hepatocarcinogenesis is induced by Sp1. We treated C57BL/6J mice with DEN to induce hepatocarcinogenesis. As shown in Figure 5(a), the livers of DEN-treated mice show pronounced hepatic steatosis and poorly differentiated tumor cells with increased mitotic figures. The protein expression of FGF21 in the liver of DEN-administrated mice was markedly increased (Figure 5(b)). Meanwhile, we detected that the protein levels of Sp1 were increased in the liver of DEN-induced hepatocarcinogenesis. And this is consistent with previous study which reported that Sp1 shows a high expression in hepatocellular carcinoma versus paired nontumor liver tissues [22]. To prove that Sp1 can induce FGF21 expression in hepatocarcinogenesis, we performed ChIP assay in the DEN-treated and control liver tissues. Our results demonstrated that the binding activity of Sp1 and FGF21 promoter DNA was increased in DEN-treated liver tissues compared with the control group (Figure 5(c)).

## 4. Discussion

FGF21 possesses multiple beneficial effects on lipid and energy homeostasis in an endocrine fashion in the liver, whereas in an autocrine or paracrine manner in the WAT [23], the molecular mechanisms underlying the transcriptional regulation of mouse FGF21 expression remain incompletely understood. In this study, we characterized the mouse FGF21 promoter region and analyzed potential transcriptional factors involved in the proximal region of FGF21 promoter. Our results demonstrate that the Sp1-binding site in the core promoter region is essential for the FGF21 transcription, and Sp1 functions as a transcriptional activator to regulate FGF21 gene expression in hepatocytes and adipocytes. Furthermore, we demonstrated that Sp1 contributes to the obesity-induced FGF21 upregulation in mouse adipose tissue and hepatic FGF21 upregulation in hepatocarcinogenesis.

Sp1 is ubiquitously expressed mammalian transcription factor involved in the regulation of many genes in response to physiological and pathological processes [24]. Oleaga et al. have reported that human FGF21 might be a Sp1 target gene by functional genomics [17]. However, experiments in their study were performed in human Hela cells and its aim was to find novel Sp1 targets involved in proliferation and cancer, and the specific Sp1 binding sites in mouse FGF21 promoter and its transcriptional role in liver and in adipose tissues, two predominant tissues of FGF21 expression, remained unclear. Therefore, our study aimed to investigate the basal transcriptional regulation of mouse FGF21 gene and focused on the underlying mechanisms accounting for the upregulation of FGF21 expression. We reported that Sp1 positively regulates the transcription of FGF21 in HepG2 cells as well as in 3T3-L1 preadipocytes. EMSA and ChIP confirmed that Sp1 specifically bound to the promoter region of FGF21 in the liver and adipose tissue. Sp1 binds to the G-rich sequences and activates or represses target genes expression [25]. In the context of the FGF21 promoter, Sp1 seems to be a positive regulator as overexpression of Sp1 increased, and suppression of Sp1 level reduced FGF21 promoter activity, respectively. Sequence analysis revealed the presence of two putative Sp1-binding sites, namely, Sp1-A and Sp1-B, in the core promoter of FGF21. Interestingly, mutation of Sp1-B but not Sp1-A exhibited a significant influence on FGF21 promoter activity during overexpression of Sp1 or suppression of Sp1 by siRNA, which we speculate most probably due to the lower DNA-binding affinity of Sp1-A compared to that of Sp1-B. The previous study demonstrated that Sp1 bound to the CT-box (5′-GGGGAGGGGC-3′) with about threefold reduced affinity in comparison with the GC-box (5′-GGGGCGGGGC-3′) [25]. Coincidentally, the sequence of Sp1-A is similar to CT-box, while that of Sp1-B is similar to GC-box. Furthermore, EMSA and ChIP assay also revealed the specific binding of Sp1 to the Sp1-B site in the liver as well as in adipose tissue. Collectively, our results suggest that Sp1 is essential for the basal transcription of FGF21 gene in hepatocytes and adipocytes.

FGF21 is expressed predominantly in the liver, adipose tissue, and pancreas, while most circulating FGF21 is derived by liver [26]. FGF21 mediates its endocrine and autocrine/paracrine actions of hormones on target tissues through FGF receptors complexed with *β*-klotho [3–5]. In liver, FGF21 stimulates gluconeogenesis, fatty acid oxidation, and ketogenesis. In adipose tissue, FGF21 regulates glucose uptake, lipolysis, and mitochondrial oxidative capacity [23]. Study from Dutchak et al. reported that FGF21 is a key mediator of the physiologic and pharmacologic actions of PPAR*γ*, as FGF21 knockout (KO) mice display defects in PPAR*γ* signaling including decreased body fat and attenuation of PPAR*γ*-dependent gene expression [10]. However, Adams et al. reported that FGF21KO mice in their study retained the full physiological response to rosiglitazone, indicating that FGF21 is not required for the beneficial metabolic outcomes of rosiglitazone treatment [27]. The author of the latter study suggested that the possible reason for the difference in phenotype between the FGF21KO lines may be the breeding strategy, diets employed, and the strain background. Further studies are required to resolve the controversy.

Circulating FGF21 levels have been found increased in obese rodents and humans [12, 13]. The elevated circulating FGF21 levels have been seen in the context of impaired glucose tolerance and increased accumulation of lipid in the liver. Thus, it was possible that FGF21 failed to exert its expected effects on glucose homeostasis and lipid oxidation, which led some researchers to hypothesize that obesity was an FGF21-resistant state [19]. Fisher et al. reported that obese mice display a significantly attenuated signaling response and impaired induction of FGF21 target genes when treated with FGF21. In addition, improvement in serum parameters such as the decline in glucose and free fatty acids are attenuated after FGF21 treatment of DIO mice [19]. However, Hale et al. reported that FGF21 stimulation of ERK phosphorylation in the liver and WAT was retained in ob/ob mice and was partially attenuated in DIO mice. Whole body metabolic effects of FGF21 were preserved in ob/ob and DIO mice. The rhFGF21 exerted strong beneficial metabolic effects at supraphysiological concentrations. Hale et al. hypothesized that elevated endogenous FGF21 levels likely represent a defense mechanism to combat obesity and insulin resistance and that obesity is a state of FGF21 relative deficiency [28]. The discrepancy may be related to the selection of different functional readouts for FGF21. Further studies will be required to thoroughly evaluate FGF21 sensitivity and responsiveness.

Results obtained in our study which showed elevated FGF21 mRNA expressions in the liver and in WAT of the diet-induced obese mice are consistent with previous studies [12, 13]. Interestingly, in accordance with the observation that HFD feeding significantly increases the transcription level of FGF21, Sp1 expression and its binding activity are increased correspondingly in adipose tissues of diet-induced obese mice. These suggest that Sp1 may be responsible for the elevated FGF21 expression in adipose tissue of diet-induced obesity mice. However, the increase of Sp1 expression in adipose tissue of HFD-fed mice was not observed in liver, suggesting that the upregulation of FGF21 expression in diet-induced mice by Sp1 is adipose tissue specifically. The increased hepatic FGF21 transcription in diet-induced mice could be regulated by other transcription factors such as XBP-1 described in our previous studies [29]. The induction of hepatic FGF21 expression in response to different physiological and pathological conditions was caused by not only metabolic extremes, but also tumorigenesis. There are studies that reported that liver FGF21 is upregulated in response to chemical (DEN treatment) and genetic-induced hepatocarcinogenesis [20, 21]. And the forced expression of FGF21 can delay the appearance of DEN-induced liver tumors, implying that elevated FGF21 serves to reduce the potential damaging effects on the liver imparted by the stress [21]. However, the transcriptional mechanism of FGF21 upregulation in hepatocarcinogenesis was still unknown. Here our study shows that the protein levels of FGF21 and Sp1 are both increased in the liver of DEN-treated mice. Furthermore, ChIP assay demonstrated that the binding activity of Sp1 and FGF21 promoter DNA was increased in DEN-treated liver tissues compared with the control group, indicating that Sp1 induce the upregulation of FGF21 in hepatocarcinogenesis.

In summary, our study has demonstrated that the proximal promoter of mouse FGF21 is located from −63 to −20 relative to transcription start site. The Sp1-binding sites within the core promoter region are essential for the positive regulation of FGF21 gene transcription. Our results suggest that Sp1 regulates liver and adipose tissue FGF21 expression in normal physiological states and contributes to the obesity-induced FGF21 upregulation in adipose tissue and hepatic FGF21 upregulation in hepatocarcinogenesis. As FGF21 has remarkable pharmacological effects on glucose and lipid metabolism, and potential protective effects on liver stress, our finding will provide useful information on understanding the mechanisms of the transcriptional regulation of FGF21 expression and propose a potential therapeutic strategy for the treatment of obesity and stress associated liver diseases.

## Figures and Tables

**Table 1:** Sequences of primers and oligonucleotides used for generation of constructs, mutagenesis, and EMSA probes.

Primer/oligonucleotide	Sequence (5′ → 3′)
−1432F	GGGGTACCCAAAGGTTCTCCCACGGTTC
−906F	GGGGTACCACTGCTGATACAGCTCTCCT
−472F	GGGGTACCTCCCTCAGACTCAGGAGTGC
−99F	GGGGTACCAGGTTCCTGCCAAGTGTGTCA
−63F	GGGGTACCGGAGTGGGGAGGGCACGT
−20F	GGGGTACCTGGTATTTCTGCGTTCACCAG
CR	GGAAGATCTTGGGGCTCAGGCAAAGTGAA
mSp1-A F	TACCGGAGTGGttgaGGCACGTGGGCGGGCCT
mSp1-A R	AGGCCCGCCCACGTGCCtcaaCCACTCCGGTA
mSp1-B F	AGGGCACGTGttgaGGCCTGTCTG
mSp1-B R	CAGACAGGCCtcaaCACGTGCCCT
FGF21-probe	GCACGTGGGCGGGCCTGTCTG
Sp1C-probe	GTCAAGTTCGGGGAGGGGGATCAG

Kpn I and Bgl II restriction sites are underlined. CR, common reverse primer.

Substitution mutations are represented in lowercase letters.
